# Evaluación de la calidad del informe del análisis del frotis de flujo vaginal de cinco laboratorios clínicos del Pacífico colombiano

**DOI:** 10.7705/biomedica.7551

**Published:** 2026-03-02

**Authors:** Mercedes Salcedo-Cifuentes, Javier E. Fonseca-Pérez

**Affiliations:** 1 Facultad de Salud, Universidad del Valle, Cali, Colombia Universidad del Valle Facultad de Salud Universidad del Valle Cali Colombia; 2 Mesa de Trabajo en Gestación y Reproducción, Facultad de Salud, Universidad del Valle, Cali, Colombia Universidad del Valle Facultad de Salud Universidad del Valle Cali Colombia

**Keywords:** calidad de la atención en salud, complicaciones, disbiosis, flujo vaginal, frotis vaginal, diagnóstico, vaginal discharge, dysbiosis, complications, vaginal smear, diagnosis, quality of health care

## Abstract

**Introducción.:**

El análisis del flujo vaginal cumple un papel clave en el diagnóstico clínico y en la toma de decisiones médicas. La estandarización del informe es esencial para garantizar la confiabilidad diagnóstica.

**Objetivo.:**

Evaluar la calidad del informe del frotis vaginal de cinco laboratorios clínicos de la región del Pacífico colombiano.

**Materiales y métodos.:**

Se trata de un estudio descriptivo y retrospectivo basado en los registros de cinco laboratorios (tres públicos y dos privados) ubicados en tres municipios del Pacífico colombiano. Se utilizó la guía BACOVA ERIGE para evaluar si los componentes analíticos estaban completos o incompletos. Los datos se procesaron con XLSTAT *Premium,* versión 2024, mediante cálculos de medias, desviaciones estándar, proporciones y razones. Se aplicó la prueba de X^2^ para comparar el cumplimiento entre laboratorios, y se hicieron análisis de correspondencia múltiple y análisis discriminadores para determinar los patrones del informe y las diferencias entre municipios.

**Resultados.:**

Se analizaron 1260 registros de mujeres entre los 18 y los 60 años: el 56,8 % estaban embarazadas y el 57 % se encontraban afiliadas al régimen subsidiado. El cumplimiento de la guía BACOVA ERIGE fue mayor en Ipiales (54,44 %) que en Cali (24,21 %) y Quibdó (21,35 %) (X^2^, p = 0,047). Mediante los análisis de correspondencia múltiple y discriminante, se identificaron tres diferentes patrones de informes por municipio.

**Conclusiones.:**

La variabilidad observada en los informes del frotis vaginal pone en evidencia la necesidad de estandarizar los procedimientos posteriores al análisis. La unificación de los criterios técnicos entre laboratorios mejoraría la calidad diagnóstica del desequilibrio de la flora vaginal y fortalecería la toma de decisiones clínicas.

## Abstract (en)

**Introduction.:**

Vaginal flow analysis plays a key role in clinical diagnosis and medical decision-making. Standardizing report formats remains essential to ensure diagnostic reliability.

**Objective.:**

To assess the quality of vaginal smear reporting in five clinical laboratories located in Colombia's Pacific region.

**Materials and methods.:**

This descriptive, retrospective study examined records from five laboratories (three public, two private) across three cities in the Colombian Pacific region. The evaluation followed the BACOVA ERIGE guidelines to assess the completeness of the analytical components. Researchers used XLSTAT Premium, version 2024, to compute means, standard deviations, proportions, and ratios. They applied the chi square test to compare reporting compliance across laboratories and used multiple correspondence analysis and discriminant analysis to identify reporting patterns and inter-city differences.

**Results.:**

The study analyzed 1260 records from women aged 18 to 60 years. Of these, 56.8% were pregnant, and 57% were affiliated to the subsidized healthcare system. Compliance with the BACOVA ERIGE guidelines was higher in Ipiales (54.44%) compared to Cali (24.21%) and Quibdó (21.35%) (X^2^, p = 0.047). The multiple correspondence analysis and discriminant analysis identified three distinct reporting profiles by city.

**Conclusions.:**

The variability in vaginal smear reports underscores the need to standardize post-analytical procedures. Unifying technical criteria among laboratories would improve the diagnostic quality of vaginal flora imbalance and enhance clinical decision-making.

La disbiosis vaginal, caracterizada por la pérdida de la dominancia de *Lactobacillus* sp. y el aumento de la diversidad microbiana ^1^, está relacionada con enfermedades infecciosas como la vaginosis bacteriana ^2^, aerobia ^3^ y citolítica ^4^, así como también con la candidiasis vulvovaginal ^5^. Todas las infecciones mencionadas presentan una gran prevalencia entre las mujeres en edad fértil, y generan implicaciones importantes para la salud sexual y reproductiva ^6^^,^^7^. Estas condiciones constituyen causas frecuentes de consulta ginecológica en los servicios de atención primaria, particularmente durante el embarazo, debido a que se asocian con parto prematuro, complicaciones neonatales y enfermedad inflamatoria pélvica ^8^^,^^9^.

En la práctica clínica, el frotis del flujo vaginal es un examen rutinario utilizado como herramienta diagnóstica inicial para identificar alteraciones de la microbiota vaginal. Mediante técnicas simples, este examen permite detectar células guía, estructuras micóticas, *Trichomonas* sp., *Lactobacillus* sp. y hacer recuentos de leucocitos, entre otras características del entorno vaginal ^10^.

Sin embargo, pese a su aparente sencillez, cualquier factor que comprometa la calidad del análisis o de su informe, puede generar errores relevantes en la interpretación de los resultados y afectar la toma de decisiones clínicas ^11^^,^^12^. Entre estos factores, se incluyen la falta de entrenamiento del personal, las fallas en la técnica de coloración y, especialmente, la variabilidad en la forma de informar los hallazgos. Dicha variabilidad se manifiesta en el uso de formatos no uniformes, como símbolos ("+", "++", "+++"), descripciones subjetivas ("escaso", "moderado", abundante") o la falta del recuento leucocitario por campo (a 40X o 100X), lo que dificulta la comparación entre los informes y compromete su confiabilidad diagnóstica ^13^.

A nivel internacional, se han desarrollado instrumentos como el manual BACOVA ERIGE ^14^, que establece criterios específicos sobre qué componentes deben reportarse y cómo describirlos en los informes de resultados del análisis del frotis de flujo vaginal, incluyendo los componentes químico, húmedo o en solución salina y de la coloración de Gram. Aunque este manual no está formalmente estandarizado fuera del país de origen, ha demostrado ser una herramienta útil para el análisis morfológico del flujo vaginal, ha mejorado la precisión diagnóstica y ha reducido la ambigüedad de los informes clínicos gracias a su enfoque accesible y sistemático ^15^.

En Colombia, existe una guía para el abordaje sindrómico de las infecciones del aparato genital, que reconoce la disbiosis como una de las principales causas del síndrome de flujo vaginal y enfatiza en la importancia de un diagnóstico preciso. Sin embargo, no establece directrices claras sobre cómo deben reportarse los resultados del análisis del frotis de flujo vaginal. Al momento de este estudio no se cuenta con forma de hacer el informe, lo que ocasiona un vacío normativo que dificulta la armonización en la forma de presentar los resultados ^16^.

Desde la gestión clínica, se reconoce que la armonización es un componente esencial para garantizar resultados coherentes y comparables en los procesos diagnósticos, como en el estudio microscópico en fresco y la coloración de Gram del frotis de flujo vaginal. A diferencia de la estandarización, que implica la aplicación uniforme de procedimientos, la armonización busca que los resultados sean clínicamente interpretables y útiles, independientemente del laboratorio que los emita. Así, el armonizar procesos críticos, como el informe de los resultados, contribuye a reducir errores clínicos derivados de informes dispares y a fortalecer la toma de decisiones médicas basadas en la evidencia ^12^.

En este contexto, surge la necesidad urgente de evaluar de manera sistemática la calidad del informe del frotis de flujo vaginal en los laboratorios clínicos del país, con el objetivo de evidenciar cuál es la magnitud de la variabilidad y en qué componentes del análisis se concentra. Esta información es esencial para que las instituciones educativas y los organismos rectores del sistema de salud, diseñen e implementen estrategias dirigidas a la armonización del informe, promoviendo así una atención más segura, equitativa y basada en la evidencia para las mujeres en edad fértil.

Este estudio tuvo como objetivo evaluar la calidad del informe del análisis del frotis de flujo vaginal en cinco laboratorios clínicos del Pacífico colombiano, utilizando como referencia los criterios establecidos en el manual BACOVA ERIGE, con el fin de determinar el grado de variabilidad presente y analizar sus implicaciones clínicas.

## Materiales y métodos

### Diseño del estudio

Se llevó a cabo un estudio descriptivo retrospectivo en cinco laboratorios clínicos (dos en Ipiales, dos en Santiago de Cali y uno en Quibdó), que incluyó los registros del análisis del frotis de flujo vaginal de mujeres entre los 18 y los 60 años.

### Criterios de inclusión y exclusión

Se incluyeron los resultados del análisis de los frotis de flujo vaginal solicitados por consulta externa y control prenatal. No se consideraron los registros procedentes del servicio de urgencias ni de hospitalización ginecológica.

### Fuente de información y sistematización de datos

Los datos se recolectaron a partir de los registros internos de los laboratorios participantes. Se incluyeron variables sociodemográficas (edad y afiliación al Sistema General de Seguridad Social en Salud) e institucionales (municipio y nivel de complejidad de la institución donde se ubicaba el laboratorio). Además, se incorporó una variable clínica relacionada con el estado de gestación.

Los resultados del frotis de flujo vaginal se organizaron conforme a las directrices del manual BACOVA ERIGE ^14^, agrupando los hallazgos en tres componentes:

1. químico: medición del pH y prueba de aminas;
2. húmedo: recuento de leucocitos por campo (10 campos a 40X), análisis semicuantitativo de *Lactobacillus* sp. (por cruces), y detección de *Trichomonas* sp., estructuras micóticas, células guía y bacterias móviles, y
3. coloración de Gram: recuento de leucocitos por campo (5 campos a 100X), análisis semicuantitativo de *Lactobacillus* sp., número de cocobacilos correspondientes a *Gardnerella* sp. o *Mobiluncus* sp., células clave y otros morfotipos relevantes.

Cada variable fue clasificada como "cumple" (1) o "no cumple" (0), según el manual de referencia. La extracción y sistematización de la información estuvo a cargo de auxiliares capacitados, quienes usaron como herramienta formularios digitales, según una guía estructurada por el equipo investigador, la cual fue desarrollada con base en el manual BACOVA ERIGE. El investigador principal verificó la calidad de los datos en una muestra aleatoria del 10 % de los registros.

### Análisis estadístico

Se evaluó la coherencia de los informes entre los laboratorios de una mismo municipio (Cali e Ipiales), lo cual permitió determinar qué tan factible era unificarlos por localidad. El procesamiento estadístico se realizó con XLSTAT *Premium,* versión 2024 ^17^. Las variables cualitativas se analizaron mediante proporciones y razones. Para las comparaciones, se utilizó la prueba de X^2^, considerando significativo un valor de p menor de 0,05.

Se diseñó un índice de calidad del registro por componente, cuyo cálculo consistió en el número de registros que cumplían los criterios, dividido por el total de registros por municipio.

Se hizo un análisis de correspondencia múltiple para identificar los patrones de cumplimiento y las relaciones entre las variables. Este tipo de análisis reduce la complejidad de los datos categóricos y permite visualizar en un plano cartesiano la proximidad relativa de las categorías, poniendo en evidencia su grado de correlación y variación. Los registros con categorías similares están más próximos, mientras que aquellos con categorías diferentes se encuentran más alejados. Cuanto más alejados estén del centro del plano cartesiano, mayor es la significancia estadística de la relación ^18^.

Para maximizar la separación de los grupos de observaciones obtenidos en el análisis de correspondencia múltiple y minimizar la variabilidad entre las observaciones de cada municipio, se hizo un análisis discriminador de los índices de calidad del registro según el municipio de origen de los registros ^19^.

### Manejo de los datos faltantes y de los sesgos de clasificación

No se excluyeron los informes con datos faltantes, ya que el objetivo del estudio era determinar la ausencia de información en los formatos de los informes de los resultados del análisis del frotis de flujo vaginal. En cuanto a los posibles sesgos de clasificación, se tomaron medidas para minimizarlos mediante el uso del manual BACOVA ERIGE como referencia técnica, el cual proporcionó criterios estandarizados que redujeron la variabilidad en la interpretación por parte del equipo de recolección y análisis.

### Consideraciones éticas

La investigación fue avalada por el Comité de Ética de la Facultad de Salud de la Universidad del Valle (acta E 029-021) y por las instituciones proveedoras de los registros, clasificándose como estudio con riesgo menor que el mínimo, conforme a la legislación colombiana.

## Resultados

Se analizaron 1260 registros de frotis de flujo vaginal. No se encontraron diferencias estadísticamente significativas entre los datos de los dos laboratorios de Ipiales ni entre los de Cali (X^2^, p = 0,057 y p = 0,059, respectivamente), lo que permitió agrupar los informes por municipio para el análisis posterior (cuadro 1).

**Cuadro 1:** Caracterización general de los laboratorios y de la fuente de las muestras de frotis de flujo vaginal analizados por municipio

Variables		Quibdó	Cali	Ipiales
Informes (n)		269	305	686
Nivel de complejidad de la IPS				
	Media	269	60	610
	Baja	0	245	76
Tipo de laboratorio clínico (n)				
	Público	269	177	609
	Privado	0	128	77
Pacientes				
	Edad promedio, años (DE)	27,7 (6,74)	25,0 (7,23)	28,7 (9,61)
	Estado de gestación [n (%)]			
	Sí	259 (20,6)	125 (9,9)	332 (26,3)
	No	10 (0,8)	180 (14,3)	354 (28,1)
Vinculación al SGSSS [n (%)]				
	Contributivo	51 (4,1)	2 (0,2)	1 (0,1)
	Subsidiado	193 (15,3)	137 (10,9)	388 (30,8)
	SISPI	7 (0,5)	126 (10)	236 (18,7)
	No afiliadas	18 (1,4)	40 (3,2)	61 (4,8)

DE: desviación estándar; IPS: Institución Prestadora de Servicios de Salud; SGSSS: Sistema General de Seguridad Social en Salud; SISPI: Sistema Indígena de Salud Propio Intercultural

El cumplimiento general de los criterios establecidos en el manual BACOVA ERIGE, fue del 54,4 % en Ipiales, 24,2 % en Cali y 21,3 % en Quibdó.

En los componentes químico y húmedo, en Cali y Quibdó se encontraron proporciones similares de datos no reportados, mientras que en Ipiales se encontró más completitud en los informes (p < 0,05). En el componente de la coloración de Gram, los laboratorios de Cali tuvieron más omisiones, en contraste con los de Quibdó e Ipiales, que tuvieron mayores cumplimientos (cuadro 2).

**Cuadro 2:** Proporción de datos no informados por componente y municipio

	Componente						
	Químico		Húmedo		Gram		
	n	%	n	%	n	%	p*
Quibdó	265	24,6	393	48,7	587	43,6	2,2e-16
Cali	420	34,4	449	49,1	1009	66,2	
Ipiales	3	0,44	14	0,7	1417	41,3	

* Prueba de X^2^

**Figura 1:**
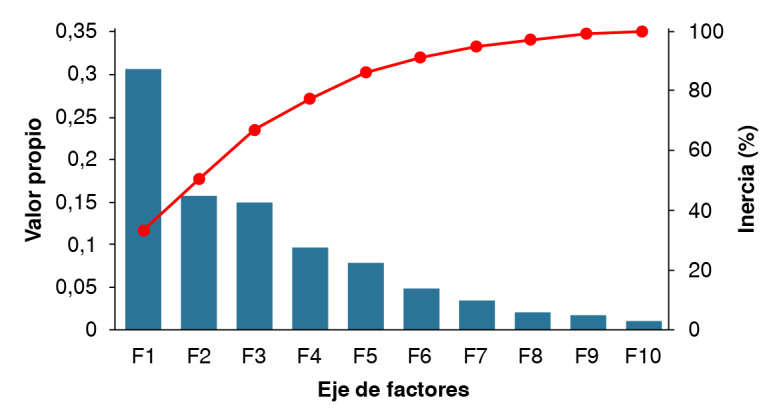
Varianza acumulada por las dimensiones factoriales del de correspondencia múltiple 100 80

En la figura 1 se muestra el aporte de cada factor a la variabilidad estructural de los datos en el análisis de correspondencia múltiple. Se seleccionaron los factores F1 y F2 para la interpretación, ya que juntos explican el 50,6 % de la varianza asociada con las categorías de los tres componentes del análisis del frotis de flujo vaginal.

En el cuadro 3 se presenta la distribución de las categorías de las variables de los dos primeros factores (F1 y F2) seleccionados según la figura 1. Se evidencia que todas las categorías relevantes (resaltadas en negrilla) aportan significativamente a estos factores. El factor 1 estuvo influido principalmente por la falta de informe de la prueba de aminas, la ausencia de *Trichomonas* sp., estructuras micóticas y células guía. El factor 2 agrupó categorías relacionadas con el informe del número de leucocitos por campo a 40X, con una distribución más equilibrada entre categorías.

**Cuadro 3:** Contribución de las categorías de las variables a los factores F1 y F2

Variable	Factor 1	Factor 2
pH, no informado	10,78	3,35
pH, informado	1,51	0,47
Prueba de aminas, ausente	10,75	7,40
Prueba de aminas, presente	7,88	5,42
Leucocitos en preparación húmeda, ausentes	0,26	37,12
Leucocitos en preparación húmeda, presentes	0,08	11,60
*Trichomonas* sp. en preparación húmeda, ausentes	17,39	5,95
*Trichomonas* sp. en preparación húmeda, presentes	4,13	1,42
Estructuras micóticas en preparación húmeda, ausentes	11,99	2,72
Estructuras micóticas en preparación húmeda, presentes	4,06	0,92
*Lactobacillus* sp. en preparación húmeda, ausentes*	0,00	0,00
*Lactobacillus* sp. en preparación húmeda, presentes*	0,00	0,00
Recuento de leucocitos en el Gram, ausentes*	5,62	1,46
Recuento de leucocitos en el Gram, presentes	0,57	0,15
Células guía en el Gram, ausentes	13,64	6,76
Células guía en el Gram, presentes	4,76	2,36
*Lactobacillus* sp. en el Gram, ausentes	1,09	0,48
*Lactobacillus* sp. en el Gram, presentes	0,49	0,22
*Mobilumcus* sp. en el Gram, ausentes	0,02	0,22
*Mobilumcus* sp. en el Gram, presentes	0,48	4,48
*Gardnerella* sp. en el Gram, ausente	1,01	1,69
*Gardnerella* sp. en el Gram, presente	3,48	5,83

Nota: las categorías relevantes están resaltadas en negrilla.

**Figura 2:**
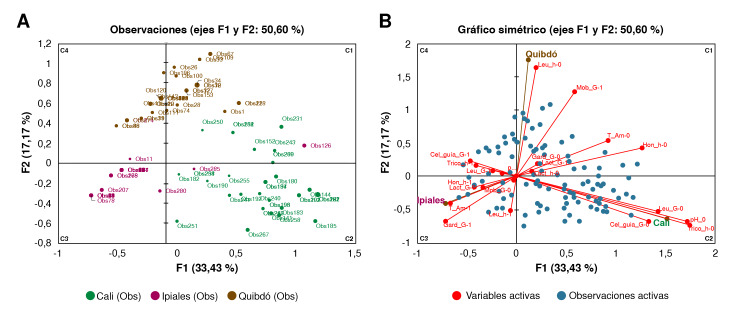
A) Representación simultánea de registros incluidos en el análisis por ciudad de origen de las muestras; B) Distribución de las categorías de las variables de estudio y registros.

La figura 2A, informes del frotis de flujo vaginal (observaciones) incluidos en el análisis de correspondencia múltiple, muestra la formación de conglomerados *(clusters)* semiestructurados, especialmente en Quibdó e Ipiales. En Cali no se presentó un conglomerado compacto; sus informes se superponen parcialmente con los de Ipiales y Quibdó, lo cual refleja una mayor variabilidad.

En la figura 2B, se presenta la distribución simultánea de los informes (observaciones) y las categorías activas de las variables consideradas en el estudio. Su distribución deja en evidencia tres conglomerados: el primero, correspondiente a Quibdó en el cuadrante 1, se caracterizó por la ausencia del informe de la prueba de aminas, del recuento de leucocitos y de estructuras micóticas. El segundo agrupa los informes de Cali con datos tanto en el cuadrante 1 como en el cuadrante 2, con omisiones similares a las de Quibdó y otras adicionales, como no informar sobre el pH, *Trichomonas* sp. y células guía. El último conglomerado, correspondiente a Ipiales, se ubicó en el cuadrante 3 y se destaca por cumplir con la prueba de aminas y el recuento de leucocitos, y con la información sobre hongos y sobre la presencia o ausencia de *Gardnerella* sp., y por no informar sobre el recuento de *Lactobacillus* sp. y *Mobiluncus* sp.

Los resultados del análisis de correspondencia múltiple sugieren que los informes de Ipiales son más completos, según los criterios de BACOVA ERIGE ^14^.

El análisis del índice de calidad del registro demostró que Cali y Quibdó tienen poca similitud en el informe del componente químico, pero gran similitud en el componente húmedo. En cuanto al componente de la coloración de Gram, Ipiales y Quibdó mostraron comportamientos similares, diferenciándose de Cali (X^2^, p = 0,049) (cuadro 4).

**Cuadro 4:** Índices de calidad de registro por componente y municipio

	Cumplimiento		
	Químico	Húmedo Coloración de Gram	Global
Cali	0,311	0,380 0,338	0,343
Ipiales	0,998	0,744 0,587	0,776
Quibdó	0,507	0,385 0,563	0,485

El índice de calidad del registro de los tres componentes presentó mucha dispersión: de 0,3 a 0,9 en el químico; de 0,3 a 0,7 en el húmedo, y de 0,3 a 0,6 en la coloración de Gram. Se evidenció el uso frecuente de escalas no estandarizadas (símbolos como "+", "++","+++" y descripciones como escaso o moderado) para informar el recuento de leucocitos. Se evidenciaron descripciones cualitativas (escaso, moderado o abundante) en el reporte de *Lactobacillus* sp., especialmente en Cali y Quibdó, lo cual incrementó la variabilidad.

**Figura 3:**
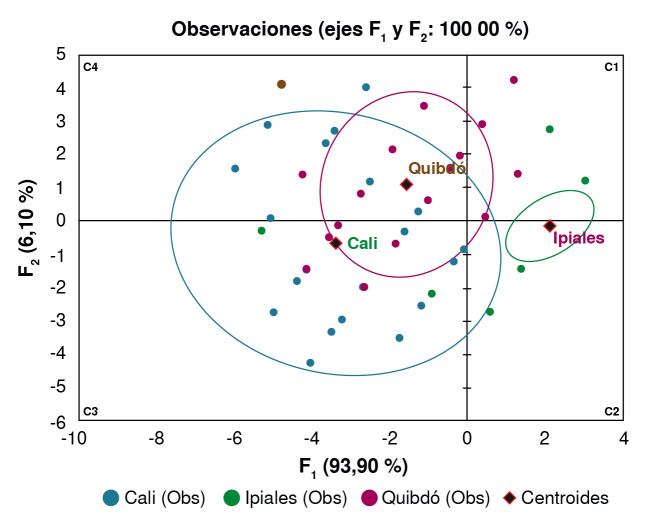
Resultados del análisis discriminante por municipio

El análisis discriminante confirmó la diferencia entre los informes de Ipiales y los de Cali y Quibdó. Aunque Quibdó y Cali se superponen en el componente húmedo -lo que significa que en este componente los resultados son muy similares, como lo evidencia el índice de calidad del registro y el análisis de correspondencia múltiple (figura 3)-, los laboratorios de Quibdó mostraron un mayor grado de cumplimiento en los componentes químico y de la coloración de Gram, alcanzando resultados similares a los de Ipiales.

## Discusión

En este estudio se evaluó la calidad del informe del análisis del frotis de flujo vaginal de cinco laboratorios clínicos de tres municipios del Pacífico colombiano, y además, se determinó el grado de variabilidad presente y se analizaron sus implicaciones clínicas. Los hallazgos revelaron una heterogeneidad significativa en el cumplimiento de los criterios del manual BACOVA ERIGE, usado como marco de referencia para el presente estudio, lo que compromete la confiabilidad de los resultados y puede afectar la toma de decisiones clínicas fundamentadas.

Más de la mitad de los informes analizados procedían de Ipiales, en comparación con Cali y Quibdó, que aportaron menos registros. En Ipiales se observó una mayor proporción de mujeres gestantes y, en términos de conteo, este subgrupo acumuló el mayor número de informes con campos sin diligenciar.

La diferencia en perfiles tiene implicaciones operativas y clínicas. En las mujeres gestantes, la disbiosis se ha asociado con parto prematuro, ruptura prematura de membranas y corioamnionitis; en mujeres no gestantes, puede propiciar el dolor genital crónico, problemas de fertilidad y mayor susceptibilidad a infecciones de transmisión sexual, incluida la infección por HIV ^20^. Además, los desequilibrios persistentes en la microbiota vaginal impactan negativamente el bienestar psicosocial ^21^.

El análisis sociodemográfico también mostró diferencias según el régimen de afiliación. En Ipiales, la mayoría estaban afiliadas al régimen subsidiado, mientras que en Cali y Quibdó predominó el régimen contributivo. Este patrón ha sido descrito en los estudios realizados en Colombia ^22^^,^^23^ y Estados Unidos ^24^, países que evidencian una mayor carga de atención y barreras en el acceso diagnóstico. Se ha observado un patrón similar en Ecuador, donde el grupo predominante estuvo conformado por mujeres entre los 21 y los 30 años, en su mayoría estudiantes o desempleadas, con baja cobertura de métodos anticonceptivos y escaso acceso a servicios especializados ^25^. Estas diferencias sugieren que el tipo de afiliación, el lugar de atención y los resultados clínicos, pueden influir en la calidad del informe del frotis de flujo vaginal, lo que resalta la necesidad de considerar estrategias orientadas a su armonización.

Los índices de calidad del registro evidenciaron que Ipiales tuvo el mejor cumplimiento. Esta diferencia se confirmó mediante el análisis de correspondencia múltiple y el análisis discriminador, que muestran que el conglomerado de Ipiales es menos disperso, en comparación con el de Quibdó y el de Cali. El acompañamiento del ente territorial de Nariño en la revisión de las instrucciones, podría explicar la mayor observancia de las recomendaciones planteadas en el manual de BACOVA ERIGE.

En el componente químico, se identificaron omisiones importantes en Cali y en Quibdó respecto al pH y la prueba de aminas, elementos esenciales en los criterios de Amsel para el diagnóstico de la vaginosis bacteriana ^26^. Solo en Ipiales se reportaron sistemáticamente estos datos, alcanzándose un índice de calidad del registro del 99,8 %, lo cual muestra que es posible aplicar criterios simples con muy buen cumplimiento.

Con respecto al componente húmedo, fue frecuente la omisión de elementos relevantes, como el recuento de leucocitos por campo, y la presencia o ausencia de *Lactobacillus* sp., estructuras micóticas, células guía y *Trichomonas* sp. Omisiones como estas comprometen la capacidad diagnóstica, dificultan el seguimiento y reducen la comparabilidad de los informes entre diferentes laboratorios clínicos ^27^.

En varios estudios recientes, se subraya la importancia de contar con formatos que especifiquen claramente qué datos se reportan de manera cuantitativa y cuáles de forma cualitativa, ya que la estandarización del informe en el examen húmedo es fundamental para garantizar interpretaciones congruentes, distinguir los cuadros infecciosos y evaluar la respuesta al tratamiento ^28^. En este contexto, se identificó el uso de escalas subjetivas como cruces ("+", "++", "+++") o términos cualitativos como "escaso", "moderado" o "abundante" para describir la presencia de leucocitos, en lugar de informar su número. En el 2023, la *International Society for the Study of Vulvovaginal Disease*^13^ señaló que esta práctica incrementa la variabilidad entre observadores y limita la utilidad clínica del informe, por lo que recomienda emplear recuentos objetivos y aplicar técnicas complementarias como la microscopía de contraste de fase.

En el componente de la coloración de Gram, se halló gran heterogeneidad en la descripción de morfotipos, lo cual refleja incumplimientos en la aplicación de criterios estandarizados, incluyendo los mencionados en el manual BACOVA ERIGE y los del sistema de puntuación de Nugent, herramienta ampliamente utilizada por los médicos tratantes como referencia diagnóstica ^2^.

El uso del análisis de correspondencia múltiple y el análisis discriminante fue un aporte metodológico novedoso en el contexto colombiano. Su incorporación en el análisis de los resultados permitió visualizar patrones de cumplimiento y grados de armonización entre laboratorios. A diferencia de una simple medición de proporciones o frecuencias, esta aproximación estadística aporta evidencia útil para auditar procesos y orientar intervenciones focalizadas. Dicha ventaja se complementa con la importancia de informar la presencia o ausencia de ciertos hallazgos, siguiendo un formato estandarizado y reproducible que facilite su interpretación clínica ^13^^,^^14^.

Las limitaciones del presente estudio incluyen su carácter exclusivamente documental y cuantitativo, sin explorar el conocimiento de bacteriólogos u homólogos sobre la información que se debe incluir en el informe del frotis de flujo vaginal ni la percepción de los médicos respecto a sus necesidades en dicho informe. Tampoco se consideraron factores institucionales, como formatos estandarizados o sistemas informáticos para la transferencia de resultados, que pueden influir en la calidad del informe. Sin embargo, los hallazgos sirven de base para futuras investigaciones mixtas que examinen la percepción de profesionales clínicos y de laboratorio, y también las barreras para implementar guías posanalíticas.

Además, estos hallazgos ofrecen insumos para que la academia, el gremio de bacteriólogos y el Instituto Nacional de Salud, avancen en la formulación de directices estandarizadas para el informe del frotis de flujo vaginal. Esto cobra especial relevancia si se considera que las trayectorias formativas del personal de laboratorio varían entre regiones y podrían influir en la calidad de los informes.

En conclusión, se evidenció una gran variabilidad en la calidad del informe del análisis del frotis del flujo vaginal entre los laboratorios evaluados. Esta heterogeneidad compromete la utilidad clínica del informe y subraya la urgencia de armonizar los criterios técnicos empleados en los diferentes componentes del análisis.

El uso de métodos estadísticos multivariados permitió identificar patrones de informe diferenciados y ofrecer una base sólida para intervenciones de mejora. Más allá del cumplimiento técnico, los hallazgos apuntan a vacíos en la estandarización posterior al análisis, y a prácticas que limitan la reproducibilidad y la interpretación de los resultados.

El fortalecimiento de la formación del talento humano, la revisión de las instrucciones institucionales y la promoción de formatos estructurados, son pasos esenciales para garantizar informes comparables y clínicamente útiles. La academia y los organismos rectores juegan un papel estratégico en este proceso, tanto en la formulación de directrices como en su incorporación en la educación y la práctica profesional.
